# Supplementary material for: Experimental Chemotherapy-Induced Mucositis: A Scoping Review Guiding the Design of Suitable Preclinical Models
Source: Int J Mol Sci. 2022 Dec 6;23(23):15434. doi: 10.3390/ijms232315434 (PMC9737148; doi:10.3390/ijms232315434)
Supplement: Supplementary file 1 [file ijms-23-15434-s001.zip › Table S2 - Summary of data collected from in vivo animal studies involving other chemotherapeutic agents-induced intestinal mucositis.pdf]

Table S2. Summary of data collected from *in vivo* animal studies involving other chemotherapeutic agents-induced intestinal mucositis. Drug concentrations obtained from studies were listed in a range of minimum to maximum dosage administered in animal models.

Drugs	Animal models	Number of articles	Dosage of drug	References
Doxorubicin	Mice	10	10 – 20 mg/kg i.p.	187, 188, 189, 190, 191, 192, 193, 8, 194, 195
	Rats	2	20 mg/kg i.p.	130, 196
	Piglets	1	100 mg/m^2 surface area i.v.	197
Capecitabine	Mice	1	500 mg/kg orally	198
Afatinib	Zebrafish	1	10 – 40 µg/g orally	199
SN38	Zebrafish	1	10 – 40 µg/g i.p.	199
Ailanthone	Mice	1	2 – 39.8 mg/kg orally	200
Melphalan	Mice	1	85.5 – 95.7 mg/m^2 i.p.	201
Busulfan	Piglets	1	12.8 mg/kg i.v.	202
	Mice	1	40 mg/kg	186
Cyclophosphamide	Mice	5	50 – 550 mg/kg i.p.	130, 203, 204, 205, 206
	Rats	1	120 mg/kg i.p.	130
	Piglets	1	120 mg/kg i.v.	202
Paclitaxel	Mice	1	2 – 4 mg/kg i.v.	207
Cytarabine (Ara-C)	Mice	1	3.6 mg/mouse i.p.	208
	Rats	1	30 mg/kg s.c.	6
Etoposide	Rats	1	40 mg/kg i.p.	130
Ifosfamide	Rabbits	1	30 – 60 mg/kg i.v.	209
Epirubicin	Mice	1	12 mg/kg i.p.	210
Dioscrin	Rats	1	60 mg/kg i.g.	148
S-1	Rats	1	20 mg/kg orally	7
	**Total**	**30**		

i.p..: intraperitoneal injection as a route of chemotherapy administration, i.v.: intravenous injection, s.c.: subcutaneous injection, i.g.: intragastrically
